# Can the Supplementation Diet With *Caryocar villosum* (Aubl.) Pers Extracts Influence the Behavioral and Metabolic Parameters of *Drosophila melanogaster*?

**DOI:** 10.1002/arch.70101

**Published:** 2025-09-25

**Authors:** Maria Eduarda Silva Soares, Andrielle Adelina Teodoro de Jesus, Douglas Lisboa Ramalho, Jonatha Flávio Souza Lemos, Diana França de Souza, Klenicy Kazumy de Lima Yamaguchi, Anderson Oliveira Souza

**Affiliations:** ^1^ Mitochondrial Metabolism and Neurotoxicology Laboratory, Department of Chemistry, Institute of Chemistry Federal University of Mato Grosso Cuiabá Brazil; ^2^ Institute of Health and Biotechnology Federal University of Amazonas Manaus Brazil

**Keywords:** Amazon plants, biochemical parameters, *Drosophila melanogaster* model, functional foods

## Abstract

Amazonian fruits, such as *Caryocar villosum*, are rich in phenolic compounds known to influence oxidative stress and mitochondrial function, suggesting a potential role in preventing age‐related diseases. This study aimed to analyze the effect of a diet supplemented with *C. villosum* on behavioral and biochemical parameters in *Drosophila melanogaster*. *D. melanogaster* were fed a diet with *C. villosum* at different concentrations from the larval stage until the fifteenth day of adulthood. A diet supplemented with 0.075 mg/mL of *C. villosum* increased mortality (*p* < 0.01) and climbing ability (*p* < 0.0001) after 10 days of feeding. However, larvae fed with 0.005–0.0125 mg/mL of *C. villosum* showed no toxic effects. Biochemical analyses revealed that ingesting 0.01 mg/mL of *C. villosum* increased activities of acetylcholinesterase (*p* < 0.01) and citrate synthase (*p* < 0.001) in head tissues. Notably, lactate levels were also elevated (*p* < 0.01) in the heads of flies fed with 0.0125 mg/mL, suggesting metabolic modulation. These results suggest that *C. villosum* supplementation for 15 days enhances neural health in *D. melanogaster*, an alternative animal model for nutrition research, through the consumption of Amazonian fruit as *C. villosum*.

## Summary

- The fruits of the Amazon represent a significant source of therapeutic agents.
- *Caryocar villosum* supplementation enhances neural health in *Drosophila melanogaster*.
- *Drosophila melanogaster* is crucial for studying neurobiological mechanisms.

## Graphical abstract

Consuming *Caryocar villosum* improves the locomotor ability of flies. The extract of *Caryocar villosum* can regulate biochemical parameters.

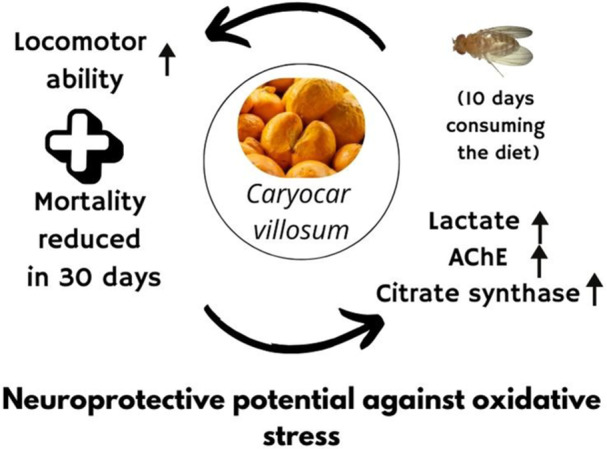

## 1 Introduction

As we age, the aging process happens naturally (Silva and Ferrari 2011). Consequently, there is a decline in certain physiological functions, which increases susceptibility to trauma, infections, and various degenerative diseases, possibly linked to the senescence of the immune system (Teixeira and Guariento 2010; Schneider and Irigaray 2008). Studies suggest that over 300 theories have been tested about aging (Farinatti 2002; Viña et al. 2007). Numerous studies have shown that aging may be associated with molecular parameters that regulate the organism's development. However, other factors, such as the production of reactive oxygen species (ROS), without the antioxidant system counterbalance, can promote the accumulation of damage or errors in the genetic material of cells, which may be linked to aging and related diseases (Viña et al. 2007; Schumacher et al. 2021).

A recent human study examined the relationship between biological aging markers and eating habits, specifically the frequent consumption of quick and easy foods, including fast food and ultra‐processed products (Cardoso et al. 2024). These foods are a significant source of carbohydrates and lipids but lack vitamins and minerals (Fostinelli et al. 2020; Ferraz et al. 2021). Conversely, proper intake of proteins and micronutrients is beneficial; certain fruits positively influence average longevity in rodents by regulating gene expression of metabolic parameters that enhance the antioxidant system (Viña et al. 2007; Ferraz et al. 2021).

The Amazon rainforest hosts a rich diversity of flora with untapped pharmacological potential, including *Caryocar villosum* (Aubl.), a fruit traditionally recognized as a “cultural marker” used for both culinary and medicinal purposes (Chisté et al. 2012). Several compounds have been identified in the fruit pulp, including phenolic compounds (gallic acid, ellagic acid) and carotenoids (lutein, antheraxanthins, zeaxanthins, and beta‐carotenes) (Chisté et al. 2012; Yamaguchi and Souza 2020), which can stabilize free radicals. Furthermore, studies indicate that it has low toxic potential (Yamaguchi et al. 2017), making it very important for the development of new drugs, as other research also confirms its antioxidant activities (Roxo et al. 2020) and anti‐inflammatory properties (Almeida et al. 2012).

To study the effects of ingesting *C. villosum*, we used *Drosophila melanogaster*, commonly known as the fruit fly, which is an easy model to manipulate and maintain (Goulart et al. 2022). Over the years, this animal model has been essential to various fields of research, including genetics, neuroscience, and neurodevelopment. This is due to its complex nervous system, composed of around 100.000 neurons. Although a fly's brain has an anatomical structure different from that of a human, many key features of the development and function of this system remain the same (Stephenson and Metcalfe 2013). Furthermore, its genome is fully sequenced and contains 75% of genes related to homologous diseases in humans (Mirzoyan et al. 2019). For this reason, it serves as an ideal model for study. Thus, we hypothesize that dietary supplementation with *C. villosum* extract modulates oxidative stress and improves behavioral and biochemical markers in *D. melanogaster*.

## 2 Material and Methods

### 2.1 Caryocar villosum Fruits

The piquiá fruits were purchased during the harvest season in January and February in the Municipality of Coari, Amazonas, middle Solimões. After acquisition, they were taken to the Organic Chemistry laboratory at the Federal University of Amazonas (UFAM) Coari campus, where they underwent a cleaning process in distilled water using a 2% sodium hypochlorite solution. They were then dried at room temperature, and measurements were taken while they were weighed on an analytical balance. The fruits were peeled and pulped manually with a knife to separate the different parts: seeds, skin, and pulp. Finally, they were crushed in a four‐knife mill (Yamaguchi et al. 2017).

The entire collection of dried fruits was stored in the Carpoteca of the Federal Institute of Amazonas—IFAM/Manaus, with registration number C1395, and registered in the Genetic Heritage System—SisGen, under registration number ADB72F4.

### 2.2 *C. villosum* Extracts

The samples were extracted by exhaust maceration using an 8:2 hydroalcoholic solvent (ethanol/water) for 48 h. The hydroalcoholic solutions were transferred to an Erlenmeyer flask, homogenized, and allowed to rest in a light‐free environment. After extraction, filtration was performed with the aid of a funnel and cotton, followed by rotary evaporation in a rotary evaporator (Fisaton) connected to a vacuum pump‐SL 061 (Solab) followed by a 12,000 mL heating blanket (Fisaton) with a controlled temperature between 20° and 30°C was used to remove the solvent. The process was concluded with freeze‐drying (LS 3000‐ Terroni) for 24 h until all the water was removed entirely. The resulting product was weighed on an analytical balance (Shimadzu) (Yamaguchi et al. 2017).

### 2.3 Chemical Characterization of *C. villosum* Extracts

Full scan and ms² data analyses were obtained using the electrospray ionization (ESI) source operating in both positive and negative modes. The samples were injected via direct insertion injection at a flow rate of 100 µL/min, within a detection range of 150–800 m/z (Yamaguchi et al. 2017).

### 2.4 Fly Strain and Rearing

All experiments in this study used *Canton Special* (wild strain) flies. The flies were raised at 25°C ± 1°C on a standard diet containing cornmeal (6.5% m/v), agar (CAS n. 9002‐18‐00) (1.0% m/v), yeast (6.5% m/v), and Nipagin (CAS n. 99‐76‐3) (3.0% v/v) (de Alencar et al. 2023; Ramalho et al. 2024).

### 2.5 Supplementation Diet to *Drosophila melanogaster*

For the treatment, various concentrations of *C. villosum* extracts were tested. Flies (both male and female) were kept under seven different experimental conditions for 3 h. During this period, eggs laid hatched, and the larvae were fed as follows: (1) A standard diet with cornmeal, agar, yeast, and nipagin (untreated flies); (2) A standard diet supplemented with a solution of *C. villosum* extract at 0.005 mg/mL; (3) A standard diet supplemented with a solution of *C. villosum* extract at 0.01 mg/mL; (4) A standard diet supplemented with a solution of *C. villosum* extract at 0.0125 mg/mL; (5) A standard diet supplemented with a solution of *C. villosum* extract at 0.0250 mg/mL; (6) A standard diet supplemented with a solution of *C. villosum* extract at 0.05 mg/mL; and (7) A standard diet supplemented with a solution of *C. villosum* extract at 0.075 mg/mL.

During treatment, both male and female flies were fed the same diet. After 15 days, all female thoraces (50 samples/microtubes) and heads (50 samples/microtubes) were dissected and homogenized using tungsten carbide beads (cat no 69997, Qiagen) in a solution containing NaCl (0.9%, pH 7.0) (CAS n. 7647‐14‐5) with a protease inhibitor diluted to 1:200 (CAS n. 66701‐25‐5). The homogenates were centrifuged at 10000 × g for 10 min at 4°C, and the supernatant was collected and stored at −20°C until use.

### 2.6 Determination of the Pupal Volume of *D. melanogaster*

After 5 days, larvae that fed on different diets begin metamorphosing into adult flies. To evaluate the effect of *C. villosum* extract samples on the volume of 40 pupae (Guan et al. 2006; Ramalho et al. 2024).

### 2.7 Survival Assay

The effect of *C. villosum* extract on the lifespan of adult flies was examined in newly emerged animals. Flies were transferred to untreated (control) and various concentrations of *C. villosum* extract starting on the first day after emergence (120 flies per vial, five vials per group). They were fed a standard diet and provided fresh food every 2 days, while maintaining optimal physiological conditions to prevent insect death caused by factors like sticking to wet food, mold, or bacterial growth (Bosco et al. 2015). Mortality was recorded in each vial over a period of 30 days (Ramalho et al. 2024).

### 2.8 Climbing Assay

The locomotor behavior of the flies was measured using a counter‐current apparatus (Simon et al. 2012). Newly emerged flies (2, 5, 10, 15, and 20 days old) were placed in empty cylindrical tubes with a height of 15 cm. The flies were then tapped to the bottom and given 60 s to climb 15 cm from the bottom of the tube. The experiment was performed across five trials/replicates. Results were obtained using the phototaxis index ∑(i*Ni)/N, where N represents the total number of *D. melanogaster* tested, and Ni represents the number of flies in the tube at the end of the experiment (Ziegler et al. 2015; Ramalho et al. 2024).

### 2.9 Lactate Content

Fifty thoraces and heads (dissected and stored at −20°C in protease inhibitor) were macerated and homogenized in 0.1 M sodium phosphate buffer (CAS n. 7601‐54‐9) at pH 7.4. The homogenate was centrifuged at 10,000×g for 10 min at 4°C, and the supernatant was collected. Lactate content was measured using the lactate oxidase method, according to manufacturer instructions (Labtest, Brazil, cat. no. #138‐1/50). Absorbance was monitored spectrophotometrically at 550 nm with a Model Cary 50MPR Varian Spectrophotometer (Varian Ltd., Melbourne, Australia). Lactate content was expressed as mg/dL of lactate per total amount of protein in the sample (Ramalho et al. 2024).

### 2.10 Protein Carbonyl

Fifty thoraces from *D. melanogaster* were homogenized in ice‐cold 100 mM Tris buffer, pH 7.4, and centrifuged at 1500 × g for 10 min at 4°C. Supernatants were separated into two fractions, resuspended with 10% trichloroacetic acid (TCA) (CAS n. 76‐03‐9), and centrifuged at 5000×g for 10 min at 4°C. One pellet was resuspended in 1 mL of 10 mM 2,4‐dinitrophenylhydrazine (DNPH) (CAS n. 119‐26‐6) in 2.5 M HCl (CAS n. 7647‐01‐0), while the other was resuspended only in 1 mL of 2.5 M HCl (Reznick and Packer 1994). After incubation (1 h, 37°C, in the dark), samples were cooled on ice for 10 min, 10% TCA was added, and centrifuged at 5000×g for 5 min at 4°C. The pellets were washed three times with 1 mL of ethanol (CAS n. 64‐17‐5)/ethyl acetate (CAS n. 141‐78‐6) (1:1) and centrifuged at 5000×g for 5 min at 4°C. The pellets were dissolved in 6 M guanidine (CAS n. 50‐01‐1) and stirred for 40 min. Protein carbonyl content was measured spectrophotometrically at 340 nm using a Model Cary 50MPR Varian Spectrophotometer (Varian Ltd., Melbourne, Australia).

### 2.11 Reduced (GSH) Glutathione Levels

Thoraces of *D. melanogaster* were homogenized in 100 mM sodium phosphate buffer (pH 7.4) containing 6 mM EDTA and 1 mg/mL OPT for 15 min (Hissin and Hilf 1976). Glutathione levels were measured spectrophotometrically at 420 nm using a Model Cary 50MPR Varian Spectrophotometer. (Varian Ltd., Melbourne, Australia). The result was reported as micrograms of GSH per milligram of protein.

### 2.12 Citrate Synthase (CS) Activity

Fifty thoraces and heads (dissected and stored at −20°C in protease inhibitor) were homogenized in Tris buffer (200 mM, pH 8.0) with Triton X‐100 (CAS n. 9036‐19‐5) at a concentration of 0.2% v/v (Spinazzi et al. 2012). The homogenates were then centrifuged at 9000×g for 30 min at 4°C, after which the supernatant was collected and the protein concentration was determined. The CS activity was initiated by adding 0.01 mg of protein to approximately 170 μL of Tris buffer containing 10 mM Acetyl‐CoA (CAS n. 32140‐51‐5), 1 mM 5’5’‐Dithiobis‐2‐nitrobenzoic acid (DTNB) (CAS n. 69‐78‐3), and 10 mM oxaloacetate (CAS n. 328‐42‐7). The reduced CoA (CoA‐SH) produced by CS activity reduces the DTNB to 2‐nitro‐5‐benzoic acid (TNB). CS activities were assessed by the rate of TNB formation, measured spectrophotometrically at 412 nm according to Srere (1969) using a Model Cary 50MPR Varian Spectrophotometer (Varian Ltd., Melbourne, Australia).

### 2.13 Acetylcholinesterase (AChE) Activity

Fifty thoraces and heads of *D. melanogaster* were homogenized in 100 mM sodium phosphate buffer (containing protease inhibitor), pH 7.4, to disrupt the cells. Homogenates were centrifuged at 9000×g for 30 min at 4°C (de Oliveira Souza et al. 2019). The supernatant (0.01 mg protein) was incubated with 100 mM sodium phosphate buffer, pH 7.4, containing 150 mM acetylthiocholine (CAS n. 1866‐15‐5) and 1 mM DTNB. AChE activity was determined spectrophotometrically using a Model Cary 50MPR Varian Spectrophotometer (Varian Ltd., Melbourne, Australia) according to the method of Ellman et al. (1961). The results were expressed as nmol conjugate formed per min per mg protein.

### 2.14 Protein Assay

The protein concentration was measured using the Bradford assay with BSA (CAS n. 9048‐46‐8) as the standard (Bradford 1976). The assay involves the interaction of the protein with the Coomassie Blue reagent (CAS n. 6104‐59‐2), and readings were taken at 596 nm.

### 2.15 Statistical Analysis

The data are presented as mean ± SEM. The number of female flies per group (n) used in each experiment. Statistical analysis was conducted using the GraphPad ©Prism version 8.0 software (San Diego, CA, USA). The statistical significance of the mean values for multiple comparisons was assessed in control and treated flies using one‐way ANOVA with the Tukey post hoc test. Results were deemed significant when *p* < 0.05 (*p < 0.05, **p < 0.01, ***p < 0.001, ****p < 0.0001) and ns for *p* > 0.05.

## 3 Results

**Figure 1:**
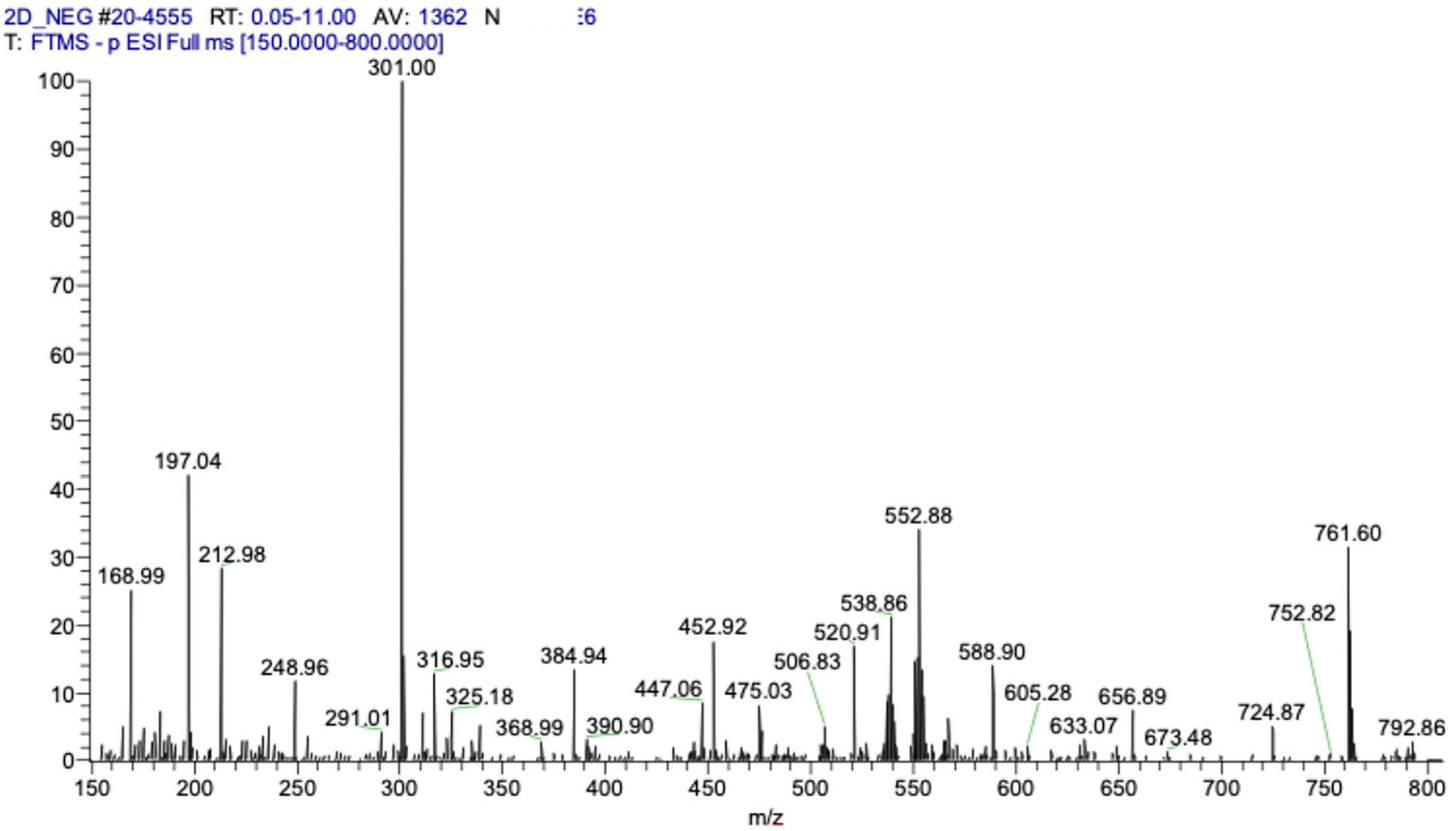
Full scan mass spectrum of the hydroalcoholic extract of *Caryocar villosum* in negative ionization mode.

Using mass spectrometry, the spectra were obtained with the electrospray source (ESI) operating in negative mode, detecting characteristic ions previously described in the literature for the species and/or genus. Among the identified ions are those with mass/charge (m/z) values of 169, 197, 291, 301, 331, 433, 447, 483, and 785 (Figure 1).

Studies by Yamaguchi et al. (2017) identified various profiles of filler masses (m/z), suggesting that these profiles contain phenolic acids, including those derived from gallic acid and ellagic acid. This finding corroborates the results observed in research involving extracts from the same species. In comparison to the fragmentation patterns found in the literature, various major ions typical of phenolics were detected in the mass spectrum [M‐H]—from the piquiá peels, as shown in Table 1.

**Table 1:** Chemical characterization of *C. villosum*.

COMPOUND	ION (M/Z)	FRAGMENTATION	REFERENCE
Galic acid	169	125	Sun et al. (2007); Yamaguchi et al. (2017)
Elagic acid	301	257, 229 and 185	Sun et al. (2007); Chisté et al. (2012); Yamaguchi et al. (2017)
Ethyl gallate	197	169 and 125	Sun et al. (2007); Ascari et al. (2013)
Brevifoline carboxylic acid	291	125	Roxo et al. (2020)
Monogaloil hexoside	331	271, 241, 211, 169, 151 e 125	Chisté et al. (2012)
Pentoside methyl elagic acid	447	301 and 299	Chisté et al. (2012)
Digaloyl‐HHDP‐glycoside	785	633, 615, 463, 419, 301, 275, 249 and 169	Roxo et al. (2020)

The development of *D. melanogaster* begins in the pupal stage, and to understand how a standard diet supplemented with hydroethanolic extract of *C. villosum* at different concentrations (0.005–0.075 mg/mL) affects it, two crucial experiments were conducted.

**Figure 2:**
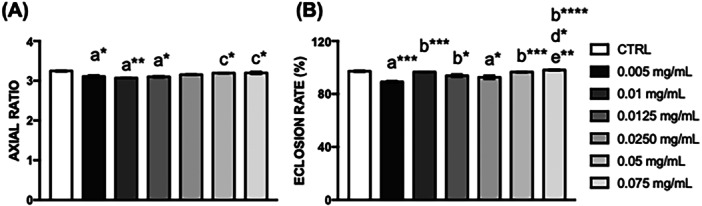
Quantification of pupal volume and hatching percentage in *D. melanogaster*. (A) The body shape of a pupa can be described by length (L) to width (W), (B) Percentage of pupae that hatched. Values represent the mean ± SEM of three experiments. The results were considered statistically significant when *p < 0.05, **p < 0.01, ***p < 0.001, ****p < 0.0001: ^a^versus CTRL, ^b^versus 0.005 mg/mL, ^c^versus 0.01 mg/mL, ^d^versus 0.0125 mg/mL, ^e^versus 0.0250 mg/mL.

The first was the pupal volume, which showed a significant reduction in the size of the pupae of flies fed with 0.01 mg/mL of hydroethanolic extract of *C. villosum* (**p < 0.01) (Figure 2A). The second showed a significant reduction in the hatching rate of flies exposed to 0.005 mg/mL (***p < 0.001) and 0.0250 mg/mL (*p < 0.05) compared with untreated flies exposed to *C. villosum* (Figure 2B).

**Figure 3:**
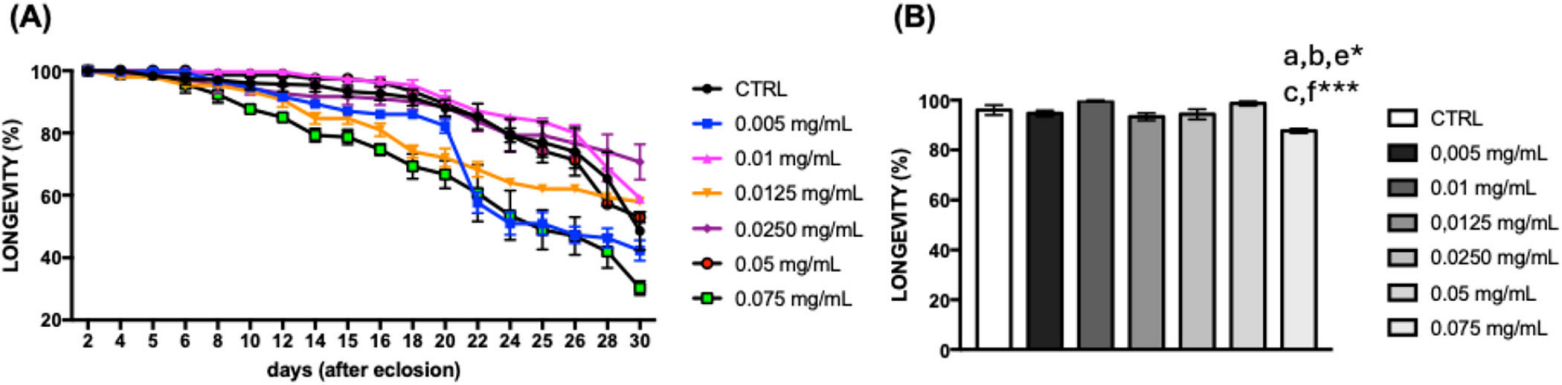
Survival rate of *D. melanogaster*. (A) Survival of flies fed a standard diet (control) or supplemented with *C. villosum* extract for 30 days and (B) statistical differences observed after 10 days of *C. villosum* extract ingestion. Statistical analyses are included as supplementary material (Figure S1). Values represent the mean ± SEM from three experiments. Results were considered statistically significant when *p < 0.05, ***p < 0.001: ^a^versus CTRL, ^b^versus 0.005 mg/mL, ^c^versus 0.01 mg/mL, ^e^versus 0.0250 mg/mL, ^f^versus 0.05 mg/mL.

The various concentrations of *C. villosum* extract added to the food significantly impacted the lifespan of *D. melanogaster* (Figure 3A). After 10 days, flies fed with 0.075 mg/mL of *C. villosum show*ed a notable decrease in longevity parameters compared to untreated animals (control) and lower concentrations of *C. villosum* (Figures 3B and S1).

**Figure 4:**
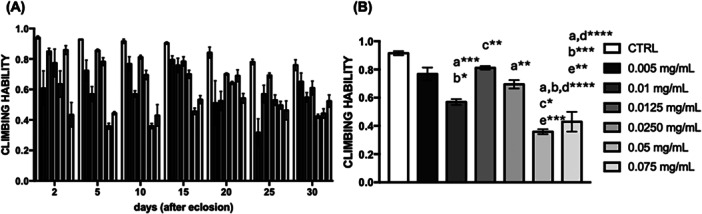
Climbing ability of *D. melanogaster*. (A) Locomotor activity of flies fed a standard diet (control) or supplemented with *C. villosum* extract for 30 days and (B) statistical differences after 10 days of ingesting *C. villosum* extract. Statistical analyses are provided as supplementary material (Figure S2). Values represent the mean ± SEM of three experiments. Results were considered statistically significant when *p < 0.05, **p < 0.01, ***p < 0.001, ****p < 0.0001: ^a^versus CTRL, ^b^versus 0.005 mg/mL, ^c^versus 0.01 mg/mL, ^d^versus 0.0125 mg/mL, ^e^vs 0.0250 mg/mL.

Flies (wild‐type strain) exhibited reduced climbing ability over 30 days (Figure 4A). However, flies given 0.01 mg/mL of *C. villosum* for 15 days demonstrated improved locomotor capacity (Figures 4B and S2).

**Figure 5:**
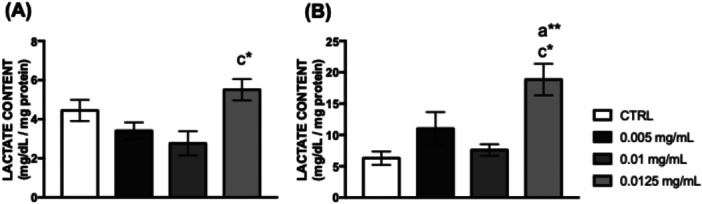
Lactate concentration in (A) the thorax and (B) the head of *D. melanogaster* supplemented with *C. villosum* extract. The values represent the mean ± SEM of three experiments. Results were considered statistically significant when *p < 0.05 and **p < 0.01: ^a^versus CTRL, ^c^versus 0.01 mg/mL.

Exposure to 0.0125 mg/mL of *C. villosum* increased lactate levels in the heads of *Drosophila melanogaster* (Figure 5B).

**Figure 6:**
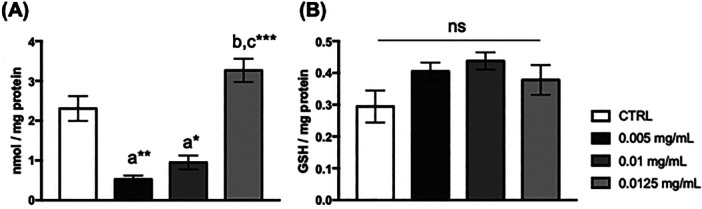
Oxidative stress parameters in the thoraces of *D. melanogaster* supplemented with *Caryocar villosum* extract. (A) Carbonyl protein and (B) reduced glutathione levels. The values represent the mean ± SEM of three experiments. The results were considered statistically significant when *p < 0.05, **p < 0.01: ^a^versus CTRL, ^b^versus 0.005 mg/mL, ^c^versus 0.01 mg/mL.

After 15 days of a diet supplemented with 0.005 and 0.01 mg/mL of *C. villosum*, carbonyl protein levels in the thoraces of flies decreased (Figure 6A). However, glutathione levels in the thoraces remained unchanged (Figure 6B).

**Figure 7:**
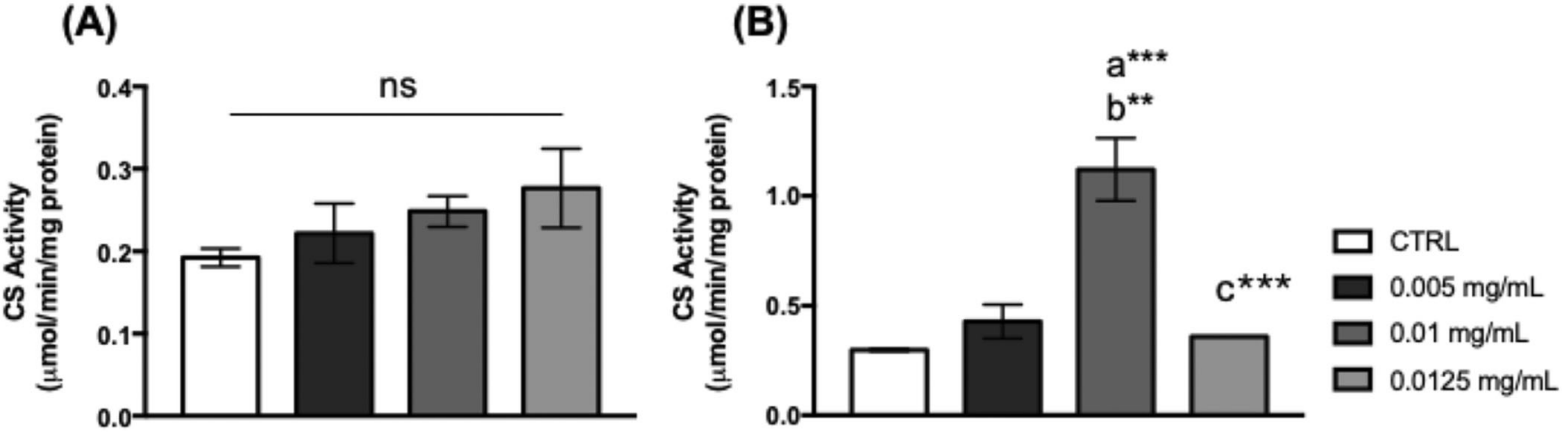
CS synthase activity in *D. melanogaster*. (A) Thorax and (B) heads of flies supplemented with *Caryocar villosum* extract. The values represent the mean ± SEM of three experiments. The results were considered statistically significant when **p < 0.01, ***p < 0.001: ^a^versus CTRL, ^b^versus 0.005 mg/mL, ^c^versus 0.01 mg/mL.

The mitochondrial metabolism of thoraces was not affected by *C. villosum* (Figure 7A). However, flies that were fed 0.01 mg/mL of *C. villosum* showed an increase in citrate synthase activity in their heads (Figure 7B).

**Figure 8:**
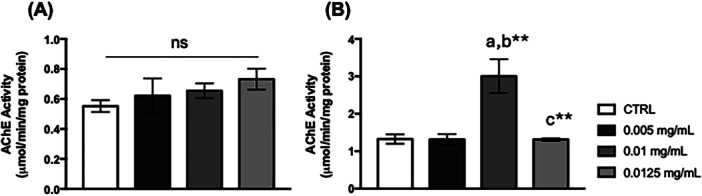
AChE activity in *D. melanogaster*. (A) Thorax and (B) Heads of flies supplemented with *Caryocar villosum* extract. The values represent the mean ± SEM of three experiments. The results were considered statistically significant when **p < 0.01: ^a^versus CTRL, ^b^versus 0.005 mg/mL, ^c^versus 0.01 mg/mL.

The cholinergic pathway controlled by acetylcholinesterase was not affected by *C. villosum* exposure in the thoraces of *D. melanogaster* (Figure 8A). Flies fed 0.01 mg/mL of *C. villosum* showed an increase in AChE activity in the heads (Figure 7B).

## 4 Discussion

Free radicals, involved in physiological signaling and regulatory processes (Prevedello and Comachio 2021), produce high levels of oxidative stress (Baptista et al. 2021). When they react with DNA, RNA, certain proteins, and other oxidizable substances, they cause damage that contributes to aging and the development of degenerative diseases such as atherosclerosis and cancer (Prevedello and Comachio 2021). Consuming an antioxidant‐rich diet or using plants for herbal remedies has become increasingly popular (Fábio et al. 2021; Viero and Dombrowski 2022). The molecules in these plants can stabilize free radicals by forming stable radical substances, which in turn reduce the risk of disease (Shen et al. 2022; Zarroug 2025).

Herein, the ESI mass spectrum of the extract from *Caryocar villosum* (Table 1) showed a predominance of the peak at m/z 301 associated with ellagic acid (Roxo et al. 2020). The fragmentation spectrum of the m/z 301 ion generated m/z 257, m/z 229, and m/z 185, indicating likely losses of the CO_2_ group (44 Da) (Sun et al. 2007; Chisté et al. 2012). In this study, we also demonstrated a peak at m/z 197 associated with ethyl galate, which has been previously described for the Caryocaraceae family, specifically in the epicarp and mesocarp of *Caryocar brasiliense* (Ascari et al. 2013; Costa Silva et al. 2025). The m/z 197 ion fragmentation spectrum produced m/z 169 and m/z 125, where the m/z 169 ions may relate to the loss of the ethyl group (28 Da) and the m/z 125 ions to CO_2_ loss (44 Da) (Sun et al. 2007). The mass spectrum [M‐H] indicated that the molecular ion m/z 291 is identified as carboxylic brevifoline acid found in the *C. villosum* bark (Roxo et al. 2020).

In *D. melanogaster*, the exposition of aspirin (Carmo et al. 2021), omega‐3 (de Oliveira Souza et al. 2017; de Oliveira Souza et al. 2019), *Duguetia furfuracea* hydroalcoholic extract (Pinho et al. 2014), saffron methanolic extract (Rao et al. 2016), honey (Cruz et al. 2016), and *Decalepis hamiltonii* extracts (Jahromi et al. 2013) demonstrated a protective effect against potential neuronal or muscular damage. Our results showed that ingesting 0.01 mg/mL *C. villosum* reduced the axial ratio while improving the eclosion rate, an essential aspect of any animal's survival. Furthermore, only 8.34% of flies fed 0.075 mg/mL of *C. villosum* for 10 days died, a similar effect compared with 0.5 mg/mL of *Lasia spinosa* stem (Men et al. 2022), 1 mg/mL of *Senecio brasiliensis* leaves (Macedo et al. 2017), 1.25 mg/mL of *Astragalus membranaceus* (Zhang et al. 2022), and 10 mg/mL of *Cyperus rotundus* (Wongchum et al. 2022).

Locomotor activity is a fundamental feature of nearly all behaving organisms (Scaplen et al. 2019). The decline of *Drosophila* climbing behavior is one of the common phenomena associated with *Drosophila* aging (Zhong et al. 2022). Our results demonstrated that in the climbing test, *D. melanogaster* fed 0.0125 mg/mL of *C. villosum* for 10 days did not experience a reduction in locomotor activity but exhibited increased carbonyl protein levels in the thorax. However, exposure to 0.05 or 0.075 mg/mL of *C. villosum* showed a significant decrease in thoracic activity, suggesting a toxic effect. Studies involving 0.1 mg/mL leaves of *Croton campestris* exhibited a protective effect on locomotor activity in *D. melanogaster* exposed to organophosphate for 2 days (Gomes et al. 2020). However, 4 mg/mL leaves of *Lippia sidoides* reduced muscular activity after 12 h of exposure (Camilo et al. 2022), suggesting that consuming molecules with antioxidant properties can improve muscular function.

Mitochondrial metabolism can be directly influenced by macro‐ or micronutrients from animal or plant sources (GÜNEŞ and Büyükgüzel 2017; Rodríguez‐Cano et al. 2020). Neuronal cells rely on glucose as their energy source, generating ATP through glycolysis, the Krebs Cycle, and electron transport chain pathways, which involve glucokinase, citrate synthase, and cytochrome oxidase enzymes, respectively. Citrate synthase plays a crucial role in the Krebs cycle for aerobic energy production by interconverting metabolites (Chhimpa et al. 2023). Our results demonstrated that flies fed 0.0125 mg/mL of *C. villosum* showed a significant increase in lactate levels in the head, a valuable biomarker of neuronal degeneration related to mitochondrial dysfunction (Cabral‐Costa et al. 2023; Ramalho et al. 2024; Ramalho et al. 2025), suggesting a neurotoxic effect caused by *C. villosum*. However, a diet supplemented with 0.01 mg/mL of *C. villosum* resulted in increased activities of citrate synthase and acetylcholinesterase. Studies indicated that high levels of citrate in mitochondria and the neuronal cytoplasm enhance acetylcholine synthesis and calcium homeostasis (Ronowska et al. 2018), a positive effect was observed after flies were fed with *C. villosum*.

The extract of *C. villosum* revealed various metabolic parameters on *D. melanogaster* fed a diet with 0.005 or 0.01 mg/mL showed no significant changes in lactate levels or the activities of CS and AChE; however, it did reduce carbonyl protein levels in the muscle. Conversely, in the head, after ingesting 0.005 or 0.01 mg/mL for 15 days, there were no significant changes in lactate levels; instead, CS and AChE activities increased, suggesting that a diet supplemented with *C. villosum* enhanced mitochondrial activity and the cholinergic system. Finally, based on this study, we propose using *D. melanogaster* for the initial screening of the potential effects of *C. villosum*. These results also encourage the continuation of this study to investigate molecules for aging treatments.

## 5 Conclusions

*D. melanogaster* fed with *C. villosum* resulted in behavioral changes, including a reduction in pupal volume and eclosion rate based on a dose–response of the diet. However, flies fed with 0.01 and 0.0125 mg/mL improved the biochemical parameters. In this study, we demonstrated, for the first time, that a diet supplemented with *C. villosum* produced significant alterations in flies, reinforcing the mitochondrial and cholinergic health effects of Amazonian fruit.

## Author Contributions

**Maria Eduarda Silva Soares:** conceptualization (equal), data curation (equal), formal analysis (equal), investigation (equal), methodology (equal), writing – original draft (equal), and review and editing (equal). **Andrielle Adelina Teodoro de Jesus:** formal analysis (equal), methodology (equal), writing – original draft (equal), and review and editing (equal). **Douglas Lisboa Ramalho:** formal analysis (equal), methodology (equal), writing – original draft (equal), and review and editing (equal). **Jonatha Flávio Souza Lemos:** formal analysis (equal), methodology (equal). **Diana França de Souza:** conceptualization (equal), data curation (equal), formal analysis (equal), investigation (equal), methodology (equal), writing – original draft (equal), and review and editing (equal). **Klenicy Kazumy de Lima Yamaguchi:** conceptualization (equal), formal analysis (equal), methodology (equal), writing – original draft (equal), and review and editing (equal). **Anderson Oliveira Souza:** conceptualization (equal), formal analysis (equal), methodology (equal), project administration (lead), writing – original draft (equal), and review and editing (equal).

## Conflicts of Interest

The authors declare no conflicts of interest.

## Supporting information


**Figure S1:** Longevity of *D. melanogaster* fed a diet supplemented with *C. villosum*. **Figure S2:** The climbing ability of *D. melanogaster* fed a diet supplemented with *C. villosum*.

## References

[arch70101-bib-0001] de Alencar, L. P. , L. L. da Costa , D. R. Lisboa , et al. 2023. “ *Piranhea trifoliata* Extracts Ameliorate Muscular Decline in *Drosophila melanogaster* Exposed to Paraquat.” Archives of Insect Biochemistry and Physiology 112: e21994. 10.1002/arch.21994. 36567513

[arch70101-bib-0002] Almeida, M. R. , J. D. C. Darin , L. C. Hernandes , et al. 2012. “Antigenotoxic Effects of Piquiá (*Caryocar villosum*) in Multiple Rat Organs.” Plant Foods for Human Nutrition 67: 171–177. 10.1007/s11130-012-0291-3. 22562095

[arch70101-bib-0003] Ascari, J. , J. A. Takahashi , and M. A. D. Boaventura . 2013. “The Phytochemistry and Biological Aspects of Caryocaraceae Family.” Revista Brasileira de Plantas Medicinais 15, no. 2: 293–308. 10.1590/S1516-05722013000200019.

[arch70101-bib-0004] Baptista, A. B. , G. N. L. Nascimento , and M. C. G. Peluzio . 2021. “ *In Vitro* Antioxidant and Microbiological Activity of *Anacardium occidentale* L. Leaf Extracts.” Revista Cereus 3: 83–98. 10.18605/2175-7275/cereus.v13n3p83-98.

[arch70101-bib-0005] Bosco, G. , M. Clamer , E. Messulam , et al. 2015. “Effects of Oxygen Concentration and Pressure on *Drosophila melanogaster*: Oxidative Stress, Mitochondrial Activity, and Survivorship.” Archives of Insect Biochemistry and Physiology 88: 222–234. 10.1002/arch.21217. 25529352

[arch70101-bib-0063] Bradford, M. M . 1976. “A Rapid and Sensitive Method for the Quantitation of Microgram Quantities of Protein Utilizing the Principle of Protein‐dye Binding.” Analytical Biochemistry 72, no. 1–2: 248–254. 10.1016/0003-2697(76)90527-3. 942051

[arch70101-bib-0006] Cabral‐Costa, J. V. , C. Vicente‐Gutiérrez , J. Agulla , et al. 2023. “Mitochondrial Sodium/Calcium Exchanger NCLX Regulates Glycolysis in Astrocytes, Impacting on Cognitive Performance.” Journal of Neurochemistry 165: 521–535. 10.1111/jnc.15745. 36563047 PMC10478152

[arch70101-bib-0007] Camilo, C. J. , D. O. D. Leite , J. W. da S. Mendes , et al. 2022. “Analysis Toxicity by Different Methods and Anxiolytic Effect of the Aqueous Extract *Lippia sidoides* Cham.” Scientific Reports 12: 20626. 10.1038/s41598-022-23999-9. 36450779 PMC9712538

[arch70101-bib-0008] Cardoso, B. R. , J. Liu , P. Machado , D. Kwon , D. W. Belsky , and E. Martinez Steele . 2024. “Association Between Ultra‐Processed Food Intake and Biological Ageing in US Adults: Findings From National Health and Nutrition Examination Survey (NHANES) 2003–2010.” Age and Ageing 53: afae268. 10.1093/ageing/afae268. 39657624 PMC11631094

[arch70101-bib-0057] do Carmo, M. K. B. , M. O. V. Figueiredo , J. M. de Souza , A. O. Souza , and C. A. C. Lima . 2021. “Neuroprotective Action of Aspirin on Paraquat Intoxication in on *Drosophila melanogaster* .” Research, Society and Development 10, no. 4: e30710414179. 10.33448/rsd-v10i4.14179.

[arch70101-bib-0009] Chhimpa, N. , N. Singh , N. Puri , and H. P. Kayath . 2023. “The Novel Role of Mitochondrial Citrate Synthase and Citrate in the Pathophysiology of Alzheimer's Disease.” Journal of Alzheimer's Disease 94: S453–S472. 10.3233/JAD-220514. PMC10473122 37393492

[arch70101-bib-0010] Chisté, R. C. , M. Freitas , A. Z. Mercadante , and E. Fernandes . 2012. “The Potential of Extracts of *Caryocar villosum* Pulp to Scavenge Reactive Oxygen and Nitrogen Species.” Food Chemistry 135, no. 3: 1740–1749. 10.1016/j.foodchem.2012.06.027. 22953916

[arch70101-bib-0011] Costa Silva, J. T. , S. A. Menezes , M. L. Mota , et al. 2025. “Antiparasitic, Antioxidant and Toxicological Activities of Methanolic Extract From the Inner Mesocarp of *Caryocar coriaceum* Wittm. (Caryocaraceae).” Pharmacological Research ‐ Natural Products 6: 100196. 10.1016/j.prenap.2025.100196.

[arch70101-bib-0012] Cruz, L. C. , A. Ecker , R. S. Dias , et al. 2016. “Brazilian Pampa Biome Honey Protects Against Mortality, Locomotor Deficits and Oxidative Stress Induced by Hypoxia/Reperfusion in Adult *Drosophila melanogaster* .” Neurochemical Research 41, no. 1–2: 116–129. 10.1007/s11064-015-1744-5. 26518676

[arch70101-bib-0062] Ellman, G. L ., K. D. Courtney , V. Andres , and R. M. Featherstone . 1961. “A new and Rapid Colorimetric Determination of Acetylcholinesterase Activity.” Biochemical Pharmacology 7, no. 2: 88–95. 10.1016/0006-2952(61)90145-9. 13726518

[arch70101-bib-0013] Fábio, F. B. F. , F. A. Wanessa , R. F. M. Mônica , et al. 2021. “Therapeutic Applications of *Caryocar brasiliense*: Systematic Review.” Journal of Medicinal Plants Research 15: 380–389. 10.5897/JMPR2021.7136.

[arch70101-bib-0014] Farinatti, P. T. V. 2002. “Teorias biológicas do envelhecimento: do genético ao estocástico.” Revista Brasileira de Medicina do Esporte 8: 129–138. 10.1590/S1517-86922002000400001.

[arch70101-bib-0015] Ferraz, I. N. , L. A. Reis , W. C. Assis , et al. 2021. “Impactos dos fatores extrínsecos no envelhecimento precoce: Uma reflexão teórica.” Research, Society and Development 10: e21210615761. 10.33448/rsd-v10i6.15761.

[arch70101-bib-0016] Fostinelli, S. , R. De Amicis , A. Leone , et al. 2020. “Eating Behavior in Aging and Dementia: The Need for a Comprehensive Assessment.” Frontiers in Nutrition 7: 604488. 10.3389/fnut.2020.604488. 33392240 PMC7772186

[arch70101-bib-0017] Gomes, K. K. , G. E. Macedo , N. R. Rodrigues , et al. 2020. “ *Croton campestris* A. St.‐Hill Methanolic Fraction in a Chlorpyrifos‐Induced Toxicity Model in *Drosophila melanogaster*: Protective Role of Gallic Acid.” Oxidative Medicine and Cellular Longevity 2020: 3960170. 10.1155/2020/3960170. 32273942 PMC7121785

[arch70101-bib-0018] Goulart, A. S. , K. M. C. Kieling , C. S. C. L. Viçosa , A. C. F. Salgueiro , and V. Folmer . 2022. “Ensino de Ciências a partir da Problematização: percepções de educandos acerca do ciclo de vida da *Drosophila melanogaster* .” Research, Society and Development 11: e31411225694. 10.33448/rsd-v11i2.25694.

[arch70101-bib-0019] Guan, X. , B. W. Middlebrooks , S. Alexander , and S. A. Wasserman . 2006. “Mutation of TweedleD, a Member of an Unconventional Cuticle Protein Family, Alters Body Shape in *Drosophila* .” Proceedings of the National Academy of Sciences 103: 16794–16799. 10.1073/pnas.0607616103. PMC1636534 17075064

[arch70101-bib-0020] Güneş, E. , and E. Büyükgüzel . 2017. “Oxidative Effects of Boric Acid on Different Developmental Stages of *Drosophila melanogaster* Meigen, 1830 (Diptera: Drosophilidae).” Turkish Journal of Entomology 41, no. 1: 3–15. 10.16970/ted.59163.

[arch70101-bib-0021] Hissin, P. J. , and R. Hilf . 1976. “A Fluorometric Method for Determination of Oxidized and Reduced Glutathione in Tissues.” Analytical Biochemistry 74, no. 1: 214–226. 10.1016/0003-2697(76)90326-2. 962076

[arch70101-bib-0022] Jahromi, S. R. , M. Haddadi , T. Shivanandappa , and S. R. Ramesh . 2013. “Neuroprotective Effect of Decalepis Hamiltonii in Paraquat‐Induced Neurotoxicity in *Drosophila melanogaster*: Biochemical and Behavioral Evidences.” Neurochemical Research 38, no. 12: 2616–2624. 10.1007/s11064-013-1179-9. 24173775

[arch70101-bib-0024] Macedo, G. E. , K. K. Gomes , N. R. Rodrigues , et al. 2017. “Senecio Brasiliensis Impairs Eclosion Rate and Induces Apoptotic Cell Death in Larvae of *Drosophila melanogaster* .” Comparative Biochemistry and Physiology. Toxicology & Pharmacology: CBP 198: 45–57. 10.1016/j.cbpc.2017.05.004. 28529177

[arch70101-bib-0025] Men, T. T. , D. T. Khang , N. T. Tuan , and D. T. X. Trang . 2022. “Anti‐Aging Effects of *Lasia spinosa* L. Stem Extract on *Drosophila melanogaster* .” Food Science and Technology 42: e38721. 10.1590/fst.38721.

[arch70101-bib-0026] Mirzoyan, Z. , M. Sollazzo , M. Allocca , A. M. Valenza , D. Grifoni , and P. Bellosta . 2019. “ *Drosophila melanogaster*: A Model Organism to Study Cancer.” Frontiers in Genetics 10: 51. 10.3389/fgene.2019.00051. 30881374 PMC6405444

[arch70101-bib-0027] de Oliveira Souza, A. , C. A. Couto‐Lima , C. H. R. Catalão , et al. 2019. “Neuroprotective Action of Eicosapentaenoic (EPA) and Docosahexaenoic (DHA) Acids on Paraquat Intoxication in *Drosophila melanogaster* .” Neurotoxicology 70: 154–160. 10.1016/j.neuro.2018.11.013. 30502405

[arch70101-bib-0028] de Oliveira Souza, A. , C. A. Couto‐Lima , M. C. Rosa Machado , E. M. Espreafico , R. G. Pinheiro Ramos , and L. C. Alberici . 2017. “Protective Action of Omega‐3 on Paraquat Intoxication in *Drosophila melanogaster* .” Journal of Toxicology and Environmental Health, Part A 80, no. 19–21: 1050–1063. 10.1080/15287394.2017.1357345. 28849990

[arch70101-bib-0029] Pinho, F. V. S. A. , G. F. Silva , G. E. Macedo , et al. 2014. “Phytochemical Constituents and Toxicity of *Duguetia furfuracea* Hydroalcoholic Extract in *Drosophila melanogaster* .” Evidence‐Based Complementary and Alternative Medicine 2014, no. 1: 838101. 10.1155/2014/838101. 25435894 PMC4243765

[arch70101-bib-0030] Prevedello, M. T. , and G. Comachio . 2021. “Antioxidantes e sua relação com os radicais livres, e Doenças Crônicas Não Transmissíveis: uma revisão de literatura/Antioxidants and Their Relationship With Free Radicals, and Chronic Non Communicable Diseases: A Literature Review.” Brazilian Journal of Development 7, no. 6: 55244–55285. 10.34117/bjdv7n6-096.

[arch70101-bib-0031] Ramalho, D. L. , J. R. Silva , M. F. Brugnera , S. Moura , and A. de Oliveira Souza . 2024. “Neurotoxic and Behavioral Deficit in *Drosophila melanogaster* Exposed to Photocatalytic Products of Paraquat.” Neurotoxicology 104: 11–19. 10.1016/j.neuro.2024.06.012. 38981577

[arch70101-bib-0032] Ramalho, D. L. , J. R. Silva , M. E. Monteiro Martins dos Santos , et al. 2025. “The Neurotoxicity of Paraquat and Its Degradation Products on *Drosophila melanogaster* .” Scientific Reports 15: 16447. 10.1038/s41598-025-86413-0. 40355491 PMC12069668

[arch70101-bib-0033] Rao, S. V. , Muralidhara , S. C. Yenisetti , and P. S. Rajini . 2016. “Evidence of Neuroprotective Effects of Saffron and Crocin in a *Drosophila* Model of Parkinsonism.” Neurotoxicology 52: 230–242. 10.1016/j.neuro.2015.12.010. 26705857

[arch70101-bib-0034] Reznick, A. Z. , and L. Packer . 1994. “Oxidative Damage to Proteins: Spectrophotometric Method for Carbonyl Assay.” Methods in Enzymology 233: 357–363. 10.1016/s0076-6879(94)33041-7. 8015470

[arch70101-bib-0035] Rodríguez‐Cano, A. M. , C. C. Calzada‐Mendoza , G. Estrada‐Gutierrez , J. A. Mendoza‐Ortega , and O. Perichart‐Perera . 2020. “Nutrients, Mitochondrial Function, and Perinatal Health.” Nutrients 12, no. 7: 2166. 10.3390/nu12072166. 32708345 PMC7401276

[arch70101-bib-0036] Ronowska, A. , A. Szutowicz , H. Bielarczyk , et al. 2018. “The Regulatory Effects of Acetyl‐CoA Distribution in the Healthy and Diseased Brain.” Frontiers in Cellular Neuroscience 12: 169. 10.3389/fncel.2018.00169. 30050410 PMC6052899

[arch70101-bib-0037] Roxo, M. , H. Peixoto , P. Wetterauer , E. Lima , and M. Wink . 2020. “Piquiá Shells (*Caryocar villosum*): A Fruit By‐Product With Antioxidant and Antiaging Properties in *Caenorhabditis elegans* .” Oxidative Medicine and Cellular Longevity 2020: 7590707. 10.1155/2020/7590707. 32908638 PMC7468659

[arch70101-bib-0038] Scaplen, K. M. , N. J. Mei , H. A. Bounds , S. L. Song , R. Azanchi , and K. R. Kaun . 2019. “Automated Real‐Time Quantification of Group Locomotor Activity in *Drosophila melanogaster* .” Scientific Reports 9, no. 1: 4427. 10.1038/s41598-019-40952-5. 30872709 PMC6418093

[arch70101-bib-0039] Schneider, R. H. , and T. Q. Irigaray . 2008. “O envelhecimento na atualidade: aspectos cronológicos, biológicos, psicológicos e sociais.” Estudos de Psicologia (Campinas) 25: 585–593. 10.1590/S0103-166X2008000400013.

[arch70101-bib-0040] Schumacher, B. , J. Pothof , J. Vijg , and J. H. J. Hoeijmakers . 2021. “The Central Role of DNA Damage in the Ageing Process.” Nature 592: 695–703. 10.1038/s41586-021-03307-7. 33911272 PMC9844150

[arch70101-bib-0041] Shen, N. , T. Wang , Q. Gan , S. Liu , L. Wang , and B. Jin . 2022. “Plant Flavonoids: Classification, Distribution, Biosynthesis, and Antioxidant Activity.” Food Chemistry 383, no. 30: 132531. 10.1016/j.foodchem.2022.132531. 35413752

[arch70101-bib-0042] Silva, W. J. M. , and C. K. B. Ferrari . 2011. “Metabolismo Mitocondrial, radicais livres e envelhecimento.” Revista Brasileira de Geriatria e Gerontologia 14: 441–451. 10.1590/S1809-98232011000300005.

[arch70101-bib-0043] Simon, A. F. , M. T. Chou , E. D. Salazar , et al. 2012. “A Simple Assay to Study Social Behavior in *Drosophila*: Measurement of Social Space Within a Group 1.” Genes, Brain and Behavior 11, no. 2: 243–252. 10.1111/j.1601-183X.2011.00740.x. 22010812 PMC3268943

[arch70101-bib-0044] Spinazzi, M. , A. Casarin , V. Pertegato , L. Salviati , and C. Angelini . 2012. “Assessment of Mitochondrial Respiratory Chain Enzymatic Activities on Tissues and Cultured Cells.” Nature Protocols 7, no. 6: 1235–1246. 10.1038/nprot.2012.058. 22653162

[arch70101-bib-0061] Srere, P. A. 1969. “[1] Citrate synthase: [EC 4.1.3.7. Citrate oxaloacetate‐lyase (CoA‐acetylating)].” Methods in Enzymology 13: 3–11. 10.1016/0076-6879(69)13005-0.

[arch70101-bib-0045] Stephenson, R. , and N. Metcalfe . 2013. “ *Drosophila melanogaster*: A Fly Through Its History and Current Use.” Journal of the Royal College of Physicians of Edinburgh 43, no. 1: 70–75. 10.4997/JRCPE.2013.116. 23516695

[arch70101-bib-0046] Sun, J. , F. Liang , Y. Bin , P. Li , and C. Duan . 2007. “Screening Non‐Colored Phenolics in Red Wines Using Liquid Chromatography/Ultraviolet and Mass Spectrometry/Mass Spectrometry Libraries.” Molecules 12, no. 3: 679–693. 10.3390/12030679. 17851421 PMC6149347

[arch70101-bib-0047] Teixeira, I. N. D. O. , and M. E. Guariento . 2010. “Biologia do envelhecimento: teorias, mecanismos e perspectivas.” Ciência & saúde coletiva 15: 2845–2857. 10.1590/S1413-81232010000600022. 20922293

[arch70101-bib-0048] Viero, A. L. C. , and P. A. Dombrowski . 2022. “Plantas medicinais e a doença de Alzheimer/Medicinal Plants and Alzheimer's Disease.” Brazilian Journal of Development 8: 16007–16021. 10.34117/bjdv8n3-033.

[arch70101-bib-0049] Viña, J. , C. Borrás , and J. Miquel . 2007. “Theories of Ageing.” IUBMB Life 59: 249–254. 10.1080/15216540601178067. 17505961

[arch70101-bib-0050] Wongchum, N. , A. Dechakhamphu , A. Ma‐Ding , et al. 2022. “The Effects of *Cyperus rotundus* L. Extracts on the Longevity of *Drosophila melanogaster* .” South African Journal of Botany 148: 218–227. 10.1016/j.sajb.2022.04.037.

[arch70101-bib-0051] Yamaguchi, K. K. L. , C. V. Lamarão , E. S. Aranha , et al. 2017. “HPLC‐DAD Profile of Phenolic Compounds, Cytotoxicity, Antioxidant and Anti‐Inflammatory Activities of the Amazon Fruit *Caryocar villosum* .” Química Nova 40: 483–490. 10.21577/0100-4042.20170028.

[arch70101-bib-0052] Yamaguchi, K. K. L. , and A. O. Souza . 2020. “Antioxidant, Hypoglycemic and Neuroprotective Activities of Extracts From Fruits Native to the Amazon Region: A Review.” Biotechnology Journal International 24, no. 6: 9–31. 10.9734/bji/2020/v24i630119.

[arch70101-bib-0053] Zarroug, S. H. O. 2025. “Caenorhabditis Elegansasin Vivomodel for the Screening of Natural Plants‐Derived Novel Anti‐Aging Compounds: A Short Introduction.” Journal of Asian Natural Products Research 27, no. 4: 577–590. 10.1080/10286020.2024.2414189. 39404185

[arch70101-bib-0054] Zhang, J. , Y. Qiao , D. Li , et al. 2022. “Aqueous Extract From Astragalus membranaceus Can Improve the Function Degradation and Delay Aging on *Drosophila melanogaster* Through Antioxidant Mechanism.” Rejuvenation Research 25, no. 4: 181–190. 10.1089/rej.2021.0081. 35726384

[arch70101-bib-0055] Zhong, L. , Z. Yang , H. Tang , Y. Xu , X. Liu , and J. Shen . 2022. “Differential Analysis of Negative Geotaxis Climbing Trajectories in *Drosophila* Under Different Conditions.” Archives of Insect Biochemistry and Physiology 111, no. 2: e21922. 10.1002/arch.21922. 35666567

[arch70101-bib-0056] Ziegler, A. B. , C. Ménagé , S. Grégoire , et al. 2015. “Lack of Dietary Polyunsaturated Fatty Acids Causes Synapse Dysfunction in the *Drosophila* Visual System.” PLoS One 10, no. 8: e0135353. 10.1371/journal.pone.0135353. 26308084 PMC4550417

