# Supplementary material for: Can the Supplementation Diet With Caryocar villosum (Aubl.) Pers Extracts Influence the Behavioral and Metabolic Parameters of Drosophila melanogaster?
Source: Arch Insect Biochem Physiol. 2025 Sep 25;120(1):e70101. doi: 10.1002/arch.70101 (PMC12464336; doi:10.1002/arch.70101)
Supplement: Supplementary file 1 — Figure S1: Longevity of D. melanogaster fed a diet supplemented with C. villosum. Figure S2: The climbing ability of D. melanogaster fed a diet supplemented with C. villosum. [file ARCH-120-e70101-s001.docx]

**Figure S1. Longevity of *D. melanogaster* fed a diet supplemented with *C. villosum*.** (A) 8th (B) 10th and (C) 12th after eclosion. The values represent the mean ± S.E.M of three independent experiments. The results were considered statistically significant when *p<0.05, **p<0.01, ***p<0.001: ^a^ *vs* CTRL, ^b^ *vs* 0.005 mg/mL, ^c^ *vs* 0.01 mg/mL, ^e^ *vs* 0.0250 mg/mL, ^f^ *vs* 0.05 mg/mL.

**Figure S2. The climbing ability of *D. melanogaster* fed a diet supplemented with *C. villosum*.** (A) 5th (B) 10th and (C) 15th after eclosion. The values represent the mean ± S.E.M of three independent experiments. The results were considered statistically significant when *p<0.05, **p<0.01, ***p<0.001, ****p<0.0001: ^a^ *vs* CTRL, ^b^ *vs* 0.005 mg/mL, ^c^ *vs* 0.01 mg/mL, ^d^ *vs* 0.0125 mg/mL, ^e^ *vs* 0.0250 mg/mL, ^f^ *vs* 0.05 mg/mL.
